# The influence of biochar on the optical phenotype and electrical signal characteristics of clustered chili pepper plants subjected to drought stress

**DOI:** 10.1080/15592324.2025.2487568

**Published:** 2025-04-04

**Authors:** Qun Yan, Bo Shi

**Affiliations:** Civil Aviation University of China, Tianjin, China

**Keywords:** Electrical signal, drought stress, biochar, optical phenotype

## Abstract

The growth state of pepper plants under different soil conditions under drought stress was studied, using RGB decomposition, thermal infrared imaging, plant electrical signal and electrochemical fingerprinting. Since porous biochar can trap more water, plants in a soil-biochar environment grow better than those in the original soil. With the increase of biochar concentration, there are more pixels in the visible image of plants, and the surface temperature of plants is lower. Biochar can also provide a stable electrochemical environment. With the increase of biochar concentration in soil, the electrical signal amplitude of pepper plants decreased and the concentration of electrochemical substances increased.

Drought is a serious environmental consequence caused by global climate change, which seriously threatens agricultural productivity. In China, drought has had a serious adverse impact on food security. The arid areas in China are mainly concentrated in the Huang-Huai-hai Plain, North China Plain and northwest China.^1^ The drought directly affected crop growth and caused a sharp decline in food production. Under drought conditions, soil moisture decreases rapidly. This impedes plant root growth and nutrient uptake, while accelerating soil erosion and degradation. In addition, drought has shortened the growing season of crops, further reducing yields.^2^

To reduce the effects of drought on plants, researchers are trying to use biochar to hold soil water. Biochar is produced by pyrolysis of materials such as crop straws at high temperatures under anaerobic or oxygen-restricted conditions.^3^ It has two key properties: first, its highly porous structure and extensive surface area give it powerful adsorption capabilities. When added to soil, biochar effectively reduces soil bulk density, enhances soil structure, and minimizes water and nutrient loss. Secondly, biochar contains a variety of trace elements that help improve soil fertility and promote crop growth.^4^ In recent years, drought has become a major challenge to crop growth and agricultural productivity. As a result, biochar has received a great deal of attention in terms of improving plant drought resistance. Most studies have shown that biochar can significantly improve soil conditions, retain water, and increase crop yields.

Dynamic monitoring is essential to understand how biochar enhances plant drought resistance. However, traditional monitoring methods often cause damage to plants, resulting in low data stability. Photophenotype and electrical signal, as nondestructive testing methods, provide a new method for monitoring plant physiological indexes.^5^ The light phenotype and electrical signal can accurately reflect the growth status and environmental conditions of the plant without harming the plant. Leaf reflectance spectrum is influenced by leaf biochemical composition and structural characteristics, and is an important tool for tracking plant physiological characteristics.^5^ Sashuang Sun^6^ used visible light and thermal imaging analysis to monitor the effects of drought on potato photosynthesis and fluorescence. Photosynthetic traits of potato were successfully estimated using image analysis. In contrast to spectral signals from plants, electrical signals are non-linear and non-stationary, with frequencies usually below 5 hz. These signals are mainly transmitted between cells and plant tissues, and their characteristics can reveal the environmental conditions and growth state of the plant.^7^ Weidong Yuan^8^ revealed the dynamic state of plants under drought conditions by studying the electrical spectra of plants. It is suggested that electrical signals can reflect the response of photosynthetic parameters to drought. Photoelectric signals have obtained rich research results in the field of plant monitoring. However, the study of photophenotype is still simple to observe the morphological changes of plants, and there is a lack of further analysis of images. The analysis of plant electrical signal and electrochemical fingerprint has not been related to plant physiological activities in detail. Therefore, the further combination of signal analysis technology and plant physiology has become a new research hotspot of plant state monitoring.

In this study, soil with different biochar content was configured. The drought environment is simulated by changing the amount of water added. The photoelectric signals of pepper plants in different soil environments under drought conditions were monitored. By correlating photoelectric phenotypic data with plant physiology, we aim to reveal the mechanisms by which biochar enhances plant drought resistance. Through interdisciplinary research, the scientific basis for rapid detection of plant drought stress by photoelectric phenotype data was established.

## 1. Materials selection

### 1.1. Material selection and cultivation

Pepper plants from Shouguang seed company, biochar from Henan Zhengzhou Xinhua Biochar company.Biochar extracted from wood is pyrolyzed at 550°C for 3 hours. Thin-skinned pepper plants are carefully selected, rinsed with deionized water to remove soil impurities, dried with absorbent paper, and then transplanted into POTS.

The soil environment includes:

Contrast: Raw soil (300 g)

Soil with 2% biochar content: (300 g soil +6 g biochar)

Soil with 4% biochar content: (300 g soil +12 g biochar)

Initially, the plants are watered thoroughly to ensure that the soil is fully saturated, allowing the plants to grow under natural conditions. After a stable growth period of 10 days, drought stress was initiated and optical phenotype data and electrical signal measurements of pepper plants were monitored. Optical phenotype and electrical signal data were recorded every two days. After 6 days of drought treatment, the plants of the experimental group showed obvious wilting phenomenon, and the experiment was ended.

## 2. Experimental testing methods

### 2.1. Plant electrical signal collection

Electrical signals are measured using an oscilloscope (OSC482) set to a sampling frequency of 2000 hz. A 1 cm long, 0.3 mm diameter stainless steel wire acts as a voltage probe and is carefully inserted into the plant tissue. The positive pole is located at the upper end of the stem, and the negative pole is located 10 mm below the positive pole. To minimize external interference, the plant is enclosed in a grounded shielding box. To ensure that the electrical signal is not affected by the stress response caused by the needle insertion, recording begins one minute after all connections are established. The amplitude of plant electrical signal is low and the ambient noise is large. Before data collection, the signal is filtered and enhanced using a voltage amplifier (AD620) (the electrical signal in the diagram is the filtered and enhanced electrical signal).

### 2.2. Plant optical phenotype data collection (visible light and thermal infrared imaging)

Visible light and thermal infrared images were captured using a Sony A210 digital camera and a Hikvision P20mas thermal infrared probe, respectively. The resolution for visible light images was 1280 × 800, while the thermal infrared images had a resolution of 256 × 192. A 60W daylight lamp was used as the light source during imaging. After capturing the images, ImageJ software was utilized for analysis, including background subtraction and RGB color decomposition of the plant’s main body images.

### 2.3. Plant electrochemical fingerprint collection

Electrochemical fingerprint measurements were conducted with a two-electrode system. Differential pulse voltammetry (DPV) was employed for scanning within a voltage range of 0 to −1.5 V, with parameters set to a pulse amplitude of 50 mV, a pulse width of 0.05 s, and a pulse period of 0.5 s. The plant was placed inside a grounded shielding box to minimize external interference, and electrochemical fingerprint testing commenced one minute after establishing the connections. All electrochemical fingerprint recordings were performed using a CS520 electrochemical workstation, and the collected data were analyzed using Origin 2021 software.

## 3. Experimental discussion

### 3.1. Analysis of plant visible light images

**Figure 1.**
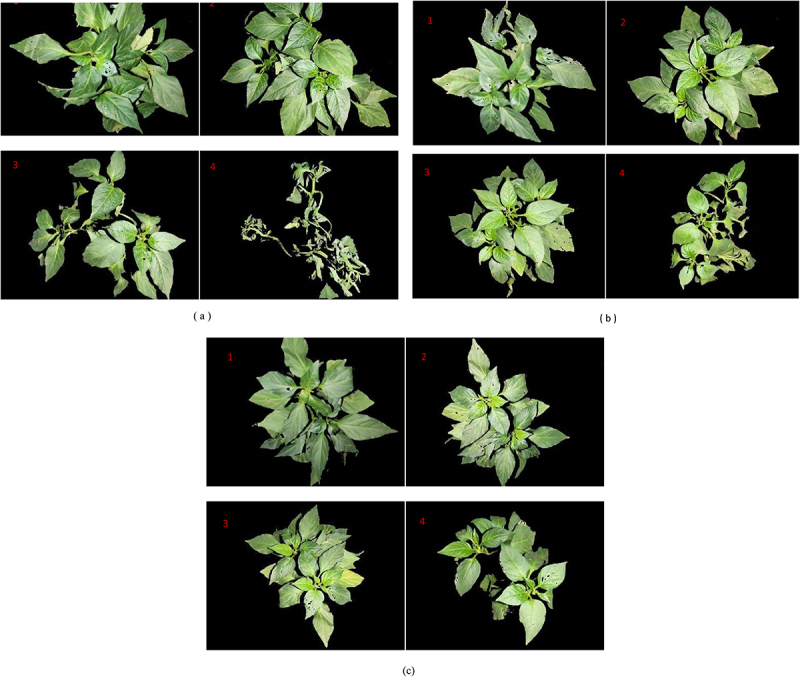
Visible light images of pepper plants in different soil environments under drought stress: (a) original soil (b) original soil + 2% biochar (c) original soil + 4% biochar. Remark: the image subscript 1,2,3,4 refers to before the drought stress，the drought lasted for 2 days, the drought lasted for 4 days, and the drought lasted for 6 days.

Figure 1 shows the visible light image of pepper seedlings under drought stress in soil environment. Under drought conditions, the leaf morphology of pepper plants changed significantly in the original soil environment (Figure 1a). The original soil environment is mainly sandy soil with large particles and large pore space.^9^ This structure results in more water loss and less moisture retention. From the second day of drought, the leaves of pepper in the original soil environment shrank and the leaf area decreased. Over time, the leaf morphology of pepper plants gradually curls or layers until they eventually wilt. This indicates that the original soil environment is not able to retain enough water to cope with drought stress.

Pepper plants in the raw soil + biochar environment grow vigorously, with dark green leaves and thick straight stems. The health of chili peppers was significantly better during the duration of the drought (Figure 1b,c). Biochar enhanced soil porosity and enhanced soil moisture retention. This gives plant roots a steady supply of water and prevents wilting due to lack of water.^10^ In addition, the porous structure of biochar and its negatively charged surface facilitate the adsorption of soil nutrients. This stabilizes the nutrients around the plant roots while minimizing nutrient loss.^11^ Visible light images showed that under drought stress, the original soil + biochar environment was more favorable to plant growth than the original soil. However, visible images could not clearly distinguish the effects of biochar concentration on drought stress resistance of pepper plants

**Figure 2.**
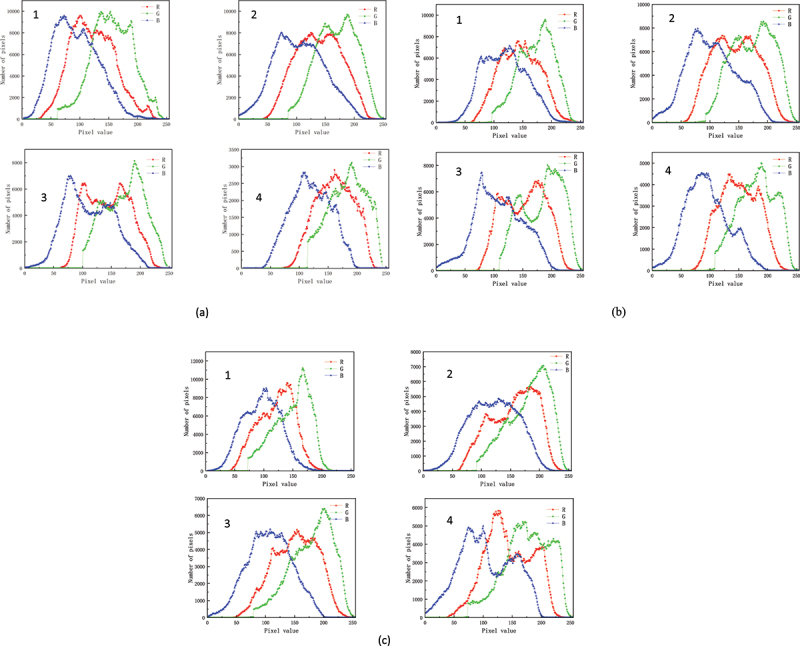
RGB decomposition of visible images of pepper plants in different soil environments under drought stress: (a) original soil (b) original soil + 2% biochar (c) original soil + 4% biochar. Remark: the image subscript 1,2,3,4 refers to before the drought stress，the drought lasted for 2 days, the drought lasted for 4 days, and the drought lasted for 6 days.

For visible images, the components in RGB and green dominance^12^ (GD) were extracted. Figure 2 shows an RGB image decomposition of a visible image of a pepper plant. Table 1 summarizes the characteristic parameters in Figure 2. It can be seen from Figure 2 and Table 1 that with the increase of drought stress duration, the pixel value of visible image of pepper in the original soil environment decreased from 915,796 to 245,341, and the half-peak width of the G-pixel peak showed a decreasing trend. This is because the original soil has limited water retention capacity, which leads to the deterioration of pepper growth and the reduction of pigment synthesis. In addition, severe water stress also accelerated the degradation of pigment.

**Table 1.** RGB decomposition feature parameters of pepper plant images in different soil environments.

		R			G			B		
		Integral area of curve	barycenter	FWHM	Integral area of curve	barycenter	FWHM	Integral area of curve	barycenter	FWHM
original soil	0 day	915796	122.18904	93.23168	915796	155.77954	84.60555	915458	94.29842	98.2579
	The second day	887883	138.79981	115.00513	887864	169.83324	96.89384	886418.5	105.1517	109.34467
	The fourth day	637264	144.67628	108.57516	637264	173.1801	91.77886	637150	111.03414	103.68584
	The sixth day	245341	161.17829	95.70155	245341	183.06305	89.72863	245341	119.85221	95.01087
original soil + 2% biochar	0 day	687753	144.34983	95.12128	687753	172.97689	75.82036	687753	118.78648	100.03365
	The second day	816240	144.50834	121.39686	816195	174.13671	109.73123	815151.5	103.79222	101.04725
	The fourth day	653911	152.72323	110.62883	653869.5	183.379	103.20266	653015	108.74241	88.57322
	The sixth day	401992	150.13314	100.86988	401970	180.50439	89.10542	401523.5	95.62488	95.62488
original soil + 4% biochar	0 day	700058	121.14169	79.02095	700058	148.35304	65.32253	700052	96.80462	80.70043
	The second day	562509	155.28203	113.66721	562498	181.07274	83.7965	562439.5	121.40066	122.98113
	The fourth day	516613	151.06924	104.62971	516585.5	179.01571	88.87418	516359	107.52033	98.9389
	The sixth day	521343.5	143.8908	112.02855	521321.5	174.94072	100.00407	520648	105.60115	124.34274

$$\mathrm{GD}=\frac{G}{R+G+B}$$

where R, G, B represent the barycenter values of the R, G, B, components

Using the data of significance analysis, the P-values were all less than 0.05.The study found that GD in the original soil environment decreased from 41.8% to 39.4%, while GD in the biochar environment remained near 41%. This indicates that biochar can effectively increase G relative content.Due to the water-storing effect of biochar, plants can still synthesize chlorophyll efficiently under drought. In the soil + biochar environment, the pixel number of visible image of pepper remained relatively stable, and the RGB pixel number did not decrease significantly with the extension of drought duration. Biochar can improve soil structure, enhance moisture retention, and stabilize nutrient supply, thereby improving its stress resistance. In drought conditions, biochar mitigates the adverse effects of water scarcity on plants, slows down the degradation of pigments, and helps maintain the green color of leaves.

### 3.2. Analysis of infrared thermal imaging of plant canopy

**Figure 3.**
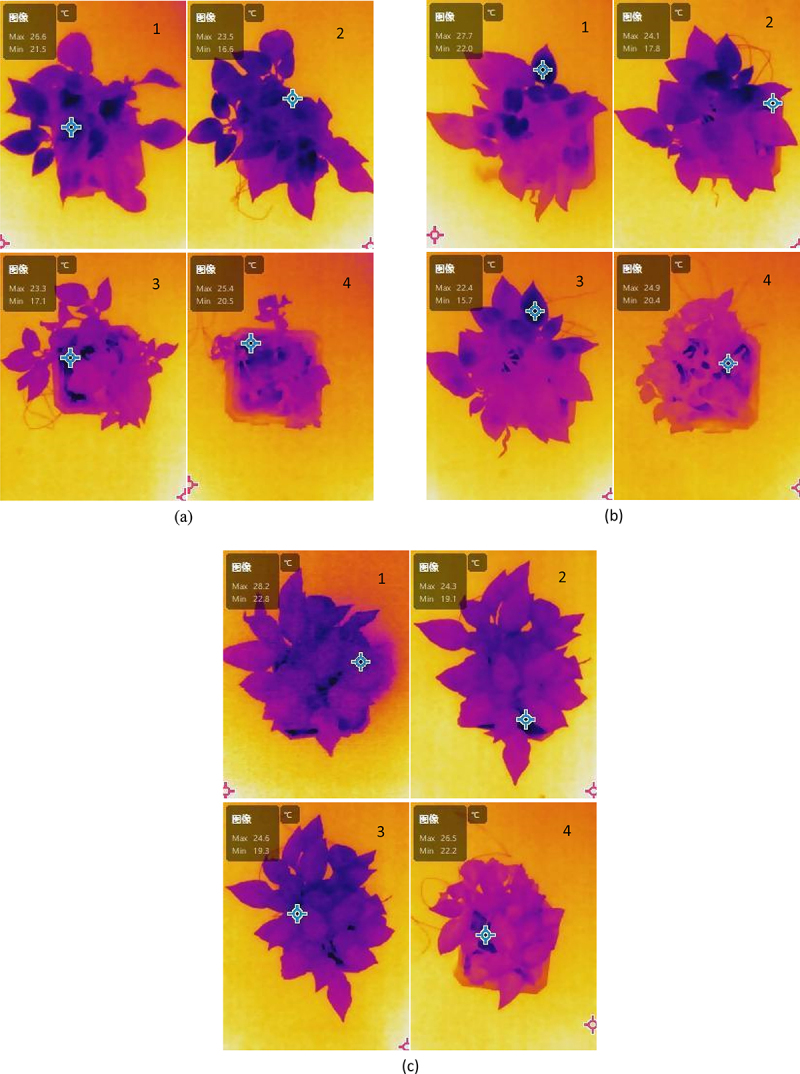
Thermal infrared images of pepper plants in different soil environments under drought stress: (a) original soil (b) original soil + 2% biochar (c) original soil + 4% biochar. Remark: the image subscript 1,2,3,4 refers to before the drought stress，the drought lasted for 2 days, the drought lasted for 4 days, and the drought lasted for 6 days.

Thermal infrared images can nondestructively monitor plant surface temperature information. Healthy plants generally dissipate heat through transpiration and maintain lower leaf temperatures, while drought-stressed plants tend to exhibit higher temperatures.^13^ As shown in Figure 3, under drought stress, with the increase of biochar content, the low temperature region of the infrared spectrum of the canopy of pepper plants significantly expanded. Biochar has a porous structure, which significantly improves the water retention capacity of the soil. Adequate moisture in the soil helps plants keep temperatures lower. In addition, biochar can enrich soil nutrient content, improve microbial activity, and promote root growth and overall plant health. These increase photosynthetic efficiency and enable plants to better regulate temperature. Therefore, the average temperature of pepper plants in soil + biochar environment was lower than that in original soil environment.

### 3.3. Plant electrical signal analysis

**Figure 4.**
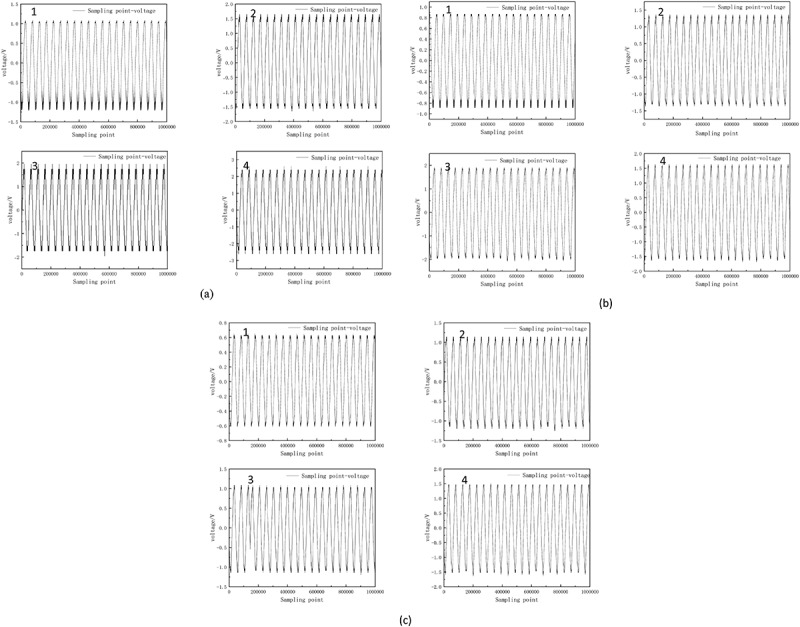
Plant electrical signal of pepper plants in different soil environments under drought stress: (a) original soil (b) original soil + 2% biochar (c) original soil + 4% biochar. Remark: the image subscript 1,2,3,4 refers to before the drought stress，the drought lasted for 2 days, the drought lasted for 4 days, and the drought lasted for 6 days.

As shown in Figure 4 and Table 2, the electrical signals of pepper plants fluctuated greatly, with a period of about 20 ms. These electrical signals come from differences in ion concentrations on the cell membrane, primarily driven by the movement of potassium (K +), sodium (Na +), calcium (Ca +), and chloride (Cl -) ions.^14^ The movement of these ions on the cell membrane is inherently unstable and is affected by various internal and external factors, resulting in fluctuations in electrical signals.With the persistence of drought stress, the peak electrical signals of pepper plants increased (as shown in Table 2, the peak electrical signals in the original soil environment increased from 2.255 V to 5.008 V; In the original soil + 2% biochar environment, it increased from 1.756 V to 3.266 V; In the original soil + 4% biochar environment, it increased from 1.214 V to 3.049 V). Water stress amplifies the plant’s electrical signals because lack of water changes the internal water potential, affecting the activity of ion channels and triggering fluctuations in electrical signals.^15^

**Table 2.** Statistical characteristics of electrical signals of pepper plants under different environments.

	curve	Maximum value	Minimum value	Peak value	cycle
Plant electrical signal curve in the original soil environment	a	1.063 V	−1.193 V	2.255 V	19.714 ms
	b	1.648 V	−1.560 V	3.208 V	20.000 ms
	c	1.744 V	−1.744 V	3.484 V	20.000 ms
	d	2.396 V	−2.612 V	5.008 V	20.000 ms
Plant electrical signal curve in the original soil + 2% biochar environment	a	0.867 V	−0.889 V	1.756 V	19.958 ms
	b	1.361 V	−1.415 V	2.776 V	20.000 ms
	c	1.851 V	−2.014 V	3.865 V	20.000 ms
	d	1.633 V	−1.633 V	3.266 V	20.000 ms
Plant electrical signal curve in the original soil + 4% biochar environment	a	0.607 V	−0.607 V	1.214 V	19.667 ms
	b	1.143 V	−1.198 V	2.341 V	20.000 ms
	c	1.034 V	−1.143 V	2.178 V	19.958 ms
	d	1.470 V	−1.579 V	3.049 V	20.000 ms

With the intensification of drought stress, the peak value of plant electrical signals in the original soil + biochar environment was significantly lower than that in the original soil system. The results indicated that the addition of biochar could effectively improve soil porosity and water retention capacity. At the end of the experiment, the peak electrical signal of pepper seedlings in the original soil + 4% biochar environment was 3.049 V, which was significantly lower than 5.009 V (original soil environment) and 3.266 V(original soil + 2% biochar environment). This indicated that the plant growth gradually improved with the increase of biochar content under drought stress. The strong adsorption and slow-release properties of biochar delay water loss and continue to provide water to plants during droughts.

**Figure 5.**
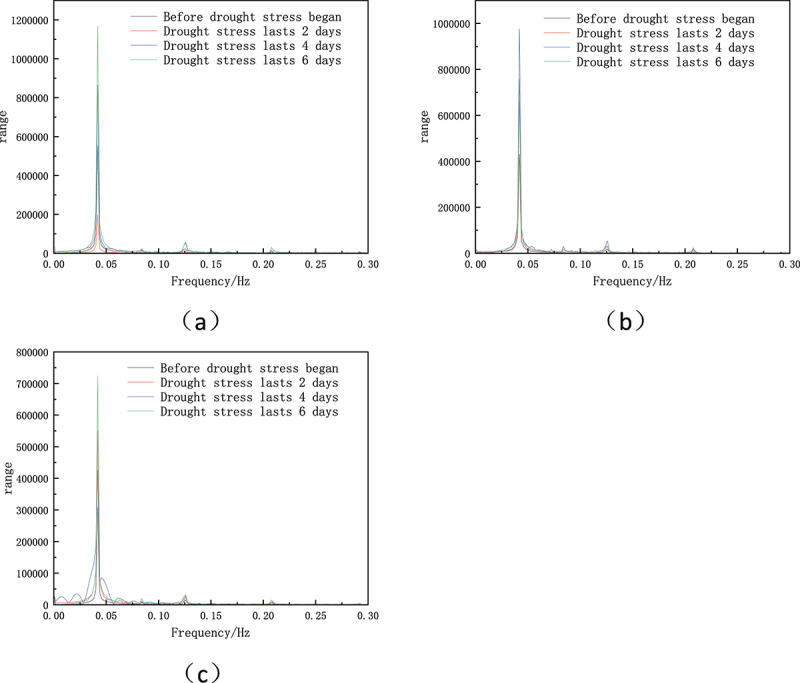
Fourier transform of electrical signals of pepper plants under different soil conditions(a) raw soil (b) raw soil + 2% biochar (c) raw soil + 4% biochar.

In order to better analyze the changes of plant electrical signals under drought stress, Fourier transform is used to decompose the signals and convert the signals from time domain to frequency domain.^16^ The method reveals various frequency components in plant electrical signals and identifies changes in specific frequency band amplitudes during drought stress. As can be seen from Figure 5, the characteristic frequency of pepper plants is 0.37 hz, and its signal peak gradually increases with the intensification of drought stress. This rise in amplitude represents the plant’s response to water stress as a mechanism for regulating water distribution and metabolic activity.

### 3.4. Plant electrochemical signal analysis

**Figure 6.**
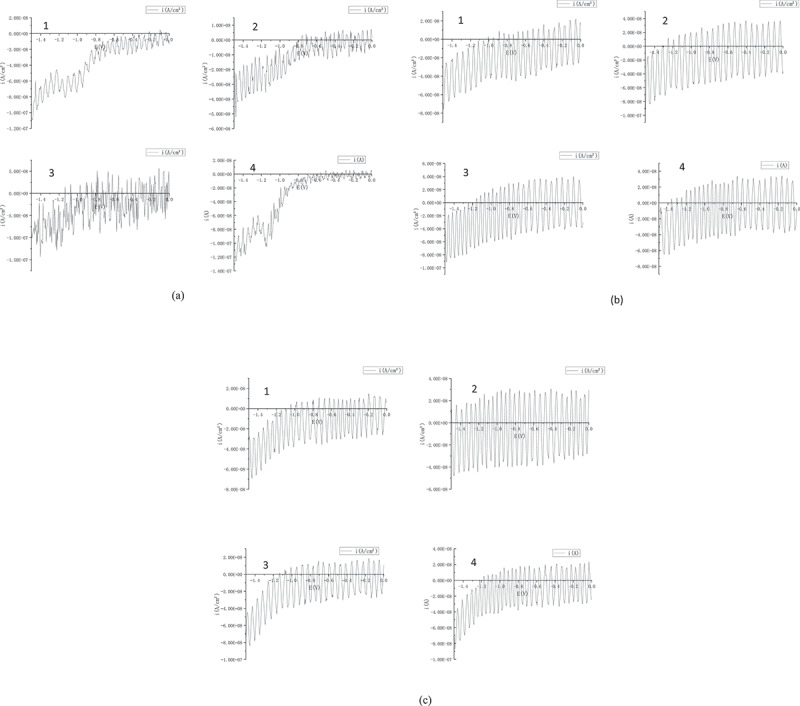
Electrochemical fingerprint curve of pepper plants under drought stress in different soil environments under drought stress: (a) original soil (b) original soil + 2% biochar (c) original soil + 4% biochar. Remark: the image subscript 1,2,3,4 refers to before the drought stress，the drought lasted for 2 days, the drought lasted for 4 days, and the drought lasted for 6 days.

Since the metal probes are made of stainless steel, the use of the positive potential scanning method may cause corrosion of the metal when measuring these substances. Therefore, the negative potential scanning method is used. As can be seen from Figure 6, Before drought stress occurs, soil moisture is sufficient, and plants can absorb sufficient water through the roots to maintain a stable distribution of electrolytes (such as potassium, sodium and calcium ions) inside and outside the cell.^17^ Due to the stable electrochemical environment, the REDOX potential peaks of various substances remain stable without significant fluctuations. Therefore, the position of the electrochemical peak in Figure 6(1) is clear and stable, without significant displacement and overlap.

As drought intensifies, DPV images of plants in the original soil environment (Figure 5(a)) become chaotic, with significant peak overlap and reduced clarity. This is due to the inability of the original soil to retain water under drought. It is difficult for plant roots to absorb enough water under drought conditions, resulting in a large amount of water loss inside the plant. The loss of water disrupts the balance of electrolytes (such as potassium, sodium, and calcium ions) in the body, resulting in an unstable electrochemical environment.^18^ The instability of electrochemical environment results in chaotic DPV images.In addition, under drought conditions, the decrease in the water content inside the plant leads to an increase in the viscosity of the cell SAP.^19^ This viscous environment promotes the formation of an adsorption layer on the electrode surface consisting of high molecular weight substances, such as proteins, carbohydrates, and phenols. When the electrode surface is covered by this adsorption layer, its electrochemical responsiveness is reduced, resulting in fuzzy peak shape.

As can be seen from Figure 5, the electrochemical fingerprint of capsicum in soil + biochar environment is more clear. In arid environments, biochar can release water that would otherwise be stored in its porous structure, thus replacing water lost by plants due to drought. This process helps stabilize the concentration of internal electrolytes, such as potassium, sodium, and calcium ions, promoting a stable and uniform electrochemical environment. With the increase of biochar content, the peak value of electrochemical fingerprint electrical signal of pepper plant increased. The peak value of current signal is proportional to the concentration of electroactive substance. These results indicated that the electroactive substance concentration of pepper plants was higher in the original soil + 4% biochar environment, and the growth was better.

## 4. Conclusion

In this study, the optical phenotype and electrical signal characteristics of pepper plants under different soil conditions under drought stress were studied by pot experiment. Because of the water retention ability of biochar, the growth performance of plants in the original soil + biochar environment is better than that in the original soil. This can be demonstrated by higher RGB pixel counts, lower temperatures, lower electrical signal amplitudes, and increased concentrations of electrochemically active substances. Under drought stress, the original soil + 4% biochar environment released more water and nutrients, and the growth performance of pepper plants was better. The electrochemical data support this. In the original soil + 4% biochar environment, the amplitude of the electrical signal is lower and the concentration of electrochemical substances is higher.

## Highlight

- Based on the voltage amplifier, the interference of stray signal to the plant electrical signal in the test process is reduced
- Plant current signals in different soil environments in arid environment were obtained by applying dynamic voltage through linear scanning using two-electrode technique.
